# Machine learning survival models trained on clinical data to identify high risk patients with hormone responsive HER2 negative breast cancer

**DOI:** 10.1038/s41598-023-35344-9

**Published:** 2023-05-26

**Authors:** Annarita Fanizzi, Domenico Pomarico, Alessandro Rizzo, Samantha Bove, Maria Colomba Comes, Vittorio Didonna, Francesco Giotta, Daniele La Forgia, Agnese Latorre, Maria Irene Pastena, Nicole Petruzzellis, Lucia Rinaldi, Pasquale Tamborra, Alfredo Zito, Vito Lorusso, Raffaella Massafra

**Affiliations:** 1 Struttura Semplice Dipartimentale di Fisica Sanitaria, I.R.C.C.S. Istituto Tumori “Giovanni Paolo II”, Viale Orazio Flacco 65, 70124 Bari, Italy; 2 Struttura Semplice Dipartimentale di Oncologia Per la Presa in Carico Globale del Paziente Oncologico “Don Tonino Bello”, I.R.C.C.S. Istituto Tumori “Giovanni Paolo II”, Viale Orazio Flacco 65, 70124 Bari, Italy; 3 Unità Operativa Complessa di Oncologia Medica, I.R.C.C.S. Istituto Tumori “Giovanni Paolo II”, Viale Orazio Flacco 65, 70124 Bari, Italy; 4 Struttura Semplice Dipartimentale di Radiologia Senologica, I.R.C.C.S. Istituto Tumori “Giovanni Paolo II”, Viale Orazio Flacco 65, 70124 Bari, Italy; 5 Unità Operativa Complessa di Anatomia Patologica, I.R.C.C.S. Istituto Tumori “Giovanni Paolo II”, Viale Orazio Flacco 65, 70124 Bari, Italy

**Keywords:** Cancer, Oncology, Risk factors

## Abstract

For endocrine-positive Her2 negative breast cancer patients at an early stage, the benefit of adding chemotherapy to adjuvant endocrine therapy is not still confirmed. Several genomic tests are available on the market but are very expensive. Therefore, there is the urgent need to explore novel reliable and less expensive prognostic tools in this setting. In this paper, we shown a machine learning survival model to estimate Invasive Disease-Free Events trained on clinical and histological data commonly collected in clinical practice. We collected clinical and cytohistological outcomes of 145 patients referred to Istituto Tumori “Giovanni Paolo II”. Three machine learning survival models are compared with the Cox proportional hazards regression according to time-dependent performance metrics evaluated in cross-validation. The c-index at 10 years obtained by random survival forest, gradient boosting, and component-wise gradient boosting is stabled with or without feature selection at approximately 0.68 in average respect to 0.57 obtained to Cox model. Moreover, machine learning survival models have accurately discriminated low- and high-risk patients, and so a large group which can be spared additional chemotherapy to hormone therapy. The preliminary results obtained by including only clinical determinants are encouraging. The integrated use of data already collected in clinical practice for routine diagnostic investigations, if properly analyzed, can reduce time and costs of the genomic tests.

## Introduction

Although there are detailed guidelines on the use of adjuvant chemotherapy, not all patients with endocrine-positive Her2 negative breast cancer at an early stage have real benefit from adding chemotherapy to adjuvant endocrine therapy. These patients are at risk for being undertreated or overtreated with endocrine therapy and chemotherapy, and tests are required to save an important number of patients from the potentially harmful side effects of chemotherapy; in particular, several studies have showed that a non-negligible proportion of BC patients, especially those with a hormone receptor-positive and lymph node-negative disease, could only be effectively treated with hormone therapy alone^1,2^. The use of adjuvant chemotherapy for estrogen receptor (ER)—positive, HER2-negative BC patients has been investigated by an impressive number of studies aimed at measuring its efficiency in a predictive manner^3^. Such studies range from genomic tests^4,5^ to sophisticated artificial intelligence models^6^, with the purpose of describing the benefit gained by each patient undergoing a specific therapy. Recent years have witnessed the availability of several molecular tests which have received long-standing recommendations in clinical guidelines^7^. In particular, the use of gene signatures has provided a standardized reproducible and quantitative tool able to define the risk of distant recurrent for ER-positive, HER2-negative early BC. Nevertheless, the adoption in the clinical practice of these decisional support tools requires a careful analysis of their cost-effectiveness, because genomics tests have an important cost and not all centers are provided with laboratories performing this type of analyses. This issue is currently driving the studies aimed the achievement of the same information by means of less expensive procedure.

In general, new interdisciplinary approaches are emerging in survival analysis, which aim to analyze data commonly collected in the clinical practice and drive the therapeutic choices. Indeed, in clinical practice, medical oncologists are increasingly using prediction tools available online, such as PREDICT, Adjuvant!, and CancerMath to guide systemic adjuvant treatment^8^. The online tools provide personalized 10-year overall survival estimates for the adjuvant treatment setting by basing their predictions on patient data (e.g. age) and tumour characteristics (e.g. size, nodal status, ER-status and grade), but they perform well at the population level, but exhibit a high degree of discordance in the intermediate and poor prognosis groups^9,10^. Furthermore, some models have been proposed for the estimation of disease-free survival with classic approaches^11,12^, but works aimed at predicting high-risk patients who might actually benefit from additional chemotherapy to hormone therapy is missing.

A wide variety of techniques is currently available, ranging from classical non-parametric Kaplan–Meier descriptive curves to extensions of the semi-parametric inferential Cox model. A limit of classical algorithms is the difficulty to model high dimensionality. Recently, machine learning techniques applied to survival tasks allow to overcome this issue^13–17^. Indeed, the classical Cox regression is a parametric model based on a probabilistic estimation whose prediction performances depend on parameters associated with each feature. Therefore, the difficulty in identifying an accurate probabilistic model increases in parallel with the increase in the included number of features considered. On the contrary, machine learning survival model, such as random forest and gradient boosting survival models, are non-parametric methods whose performances depend on the size of the training set. In practice, the latter models do not impose any hypothesis on the probabilistic distribution, thus allowing to properly model nonlinearities and interaction effects in a data driven approach^18^. If, on the one hand, these limitations are pursued to achieve the explainability of black-box machine learning survival models^19–21^, on the other their overcoming guarantees higher performances^18^.

In this work, we propose a model for estimating disease-free survival with respect to invasive events for patients with endocrine-positive and HER2 negative BC, which are potentially candidates for genomic testing, if only hormone therapy is carry out. Our preliminary study is configured to identify low- and high-risk patients and assess the chance of achieving comparable performances with genomic test but exploiting much cheaper and already available clinical data. Three machine learning survival models are compared with the Cox proportional hazards regression according to time-dependent classification performance metrics^22^. Once the best machine learning survival model was chosen, we evaluated the correlation risk score obtained from our model with that of a genetic test performed on a sample of independent patients.

## Results

### Enrolled patients and features

Our dataset is composed by clinical and cytohistological outcomes of 145 patients our extracted from our database of approximately 900 patients registered for a first BC diagnosis in the period 1997–2019 and referred to Istituto Tumori “Giovanni Paolo II” in Bari (Italy). The inclusion criteria for collecting such database were: absence of primary chemotherapy for BC, ab initio non-metastatic patient. Then, according to the genomic test eligibility criteria defined by the decree of the Ministry of Health of May 2021^23^, i.e. early stage tumor, patients not at high or low risk of recurrence with hormone responsive and HER2 negative BC, 145 patients wase extracted. In line with the aim of our work, we considered the only patients who did not undergo that chemotherapy. In fact, for patients who have undergone chemotherapy, the absence of a second event could be due to a patient-specific positive prognostic profile and not necessarily to an effect of the therapeutic treatment. In other words, there may be patients who would not have relapsed even if they had not undergone additional chemotherapy.

In this work, we specifically focus our attention on breast cancer-related invasive disease events (IDEs), which include local recurrence, the appearance of distant visceral and soft tissue metastases, contralateral invasive breast cancer or a second primary tumor^24^.

Collected features were the age at diagnosis, tumor size (diameter: T1a, T1b, T1c, T2, T3, T4), histological subtype (ductal, lobular, other), type of surgery (quadrantectomy/mastectomy), estrogen receptor expression (ER, %), progesterone receptor expression (PgR, %), cellular marker for proliferation (Ki67, %), histological grade (grading, Elston–Ellis scale: G1, G2, G3), human epidermal growth factor receptor-2 score (HER2/neu: 0^+^, 1^+^, 2^+^), the number of metastatic and eradicated lymph nodes, lymph nodes dissection (no/yes), sentinel lymph node (no, negative, positive), lymph nodes stage (N: 0, 1, 2, 3), in situ component (absent, G1, G2, G3, present but not typed), lymphovascular invasion (absent, focal, extensive, present but not typed), multiplicity (no/yes) and previous tumors (no/yes). Moreover, 97.41% of the collected patients did not undergo radiotherapy, we therefore did not consider it significant to include this information in the analyses.

The data set is described in Table 1. The set of predictive features is composed by $N=18$ prognostic factors, typically considered by clinicians during the first tumor diagnosis and related surgery. The missing data recovery has been implemented by means of the Python package musingly (v. 0.2.0).

**Table 1:** Observed patients’ statistics according to considered features.

Features	Counts (%)	Features	Counts (%)
Overall	145 (100)		
Lymph nodes stage		Type of surgery	
N0	114 (78.6)	Quadrantectomy	108 (74.5)
N1	26 (17.9)	Mastectomy	37 (25.5)
N2	2 (1.4)	In situ component	
N3	1 (0.7)	Absent	101 (69.7)
NA	2 (1.4)	G1	7 (4.8)
Lymph node dissection		G2	8 (5.5)
No	34 (23.5)	G3	4 (2.8)
Yes	104 (71.7)	Present, not typed	25 (17.2)
NA	7 (4.8)	HER2/neu	
Sentinel lymph node		0^+^	70 (48.3)
No	97 (66.9)	1^+^	56 (38.6)
Negative	34 (23.5)	2^+^	15 (10.3)
Positive	8 (5.5)	NA	4 (2.8)
NA	6 (4.1)	Multiplicity	
Grading		No	118 (81.4)
G1	20 (13.8)	Yes	27 (18.6)
G2	104 (71.7)	Diameter	
G3	17 (11.7)	T1b	26 (17.9)
NA	4 (2.8)	T1c	68 (46.9)
Lymphovascular Invasion		T2	42 (29.0)
Absent	98 (67.6)	T3	1 (0.7)
Focal	33 (22.8)	T4	3 (2.1)
Extensive	4 (2.8)	NA	5 (3.5)
Present, not typed	10 (6.9)	Histologic type	
Previous tumors		Ductal	107 (73.8)
No	140 (96.6)	Lobular	20 (13.8)
Yes	5 (3.5)	Other	18 (12.4)

	Median [${q}_{0},{q}_{1},{q}_{3},{q}_{4}$]		Median [${q}_{0},{q}_{1},{q}_{3},{q}_{4}$]
ER	80 [9, 70, 90, 100]	Age at diagnosis	59 [32, 49, 67, 86]
PgR	60 [0, 20, 90, 98]	Metastatic lymph nodes	0 [0, 0, 0, 10]
Ki67	12 [1, 5, 20, 70]	Eradicated lymph nodes	16 [0, 2, 23, 40]

A separate dataset, composed by 27 patients endowed with EndoPredict® (EP) scores and undergoing surgery during 2021 in our institute, is exploited for further evaluations of our survival estimation. EP is a gene expression test for patients with ER-positive and HER2-negative early-stage BC, both node-negative and node-positive (N0, N1, micrometastasis). It is a second-generation test that combines a molecular score of 12 genes with tumor size and lymph node status^5^. This genomic test has entered the clinical practice of the Istituto Tumori 'Giovanni Paolo II' in Bari since 2021 and it is used by the Breast Care team when the clinical case is highly doubtful.

### Time dependent classification

**Figure 1:**
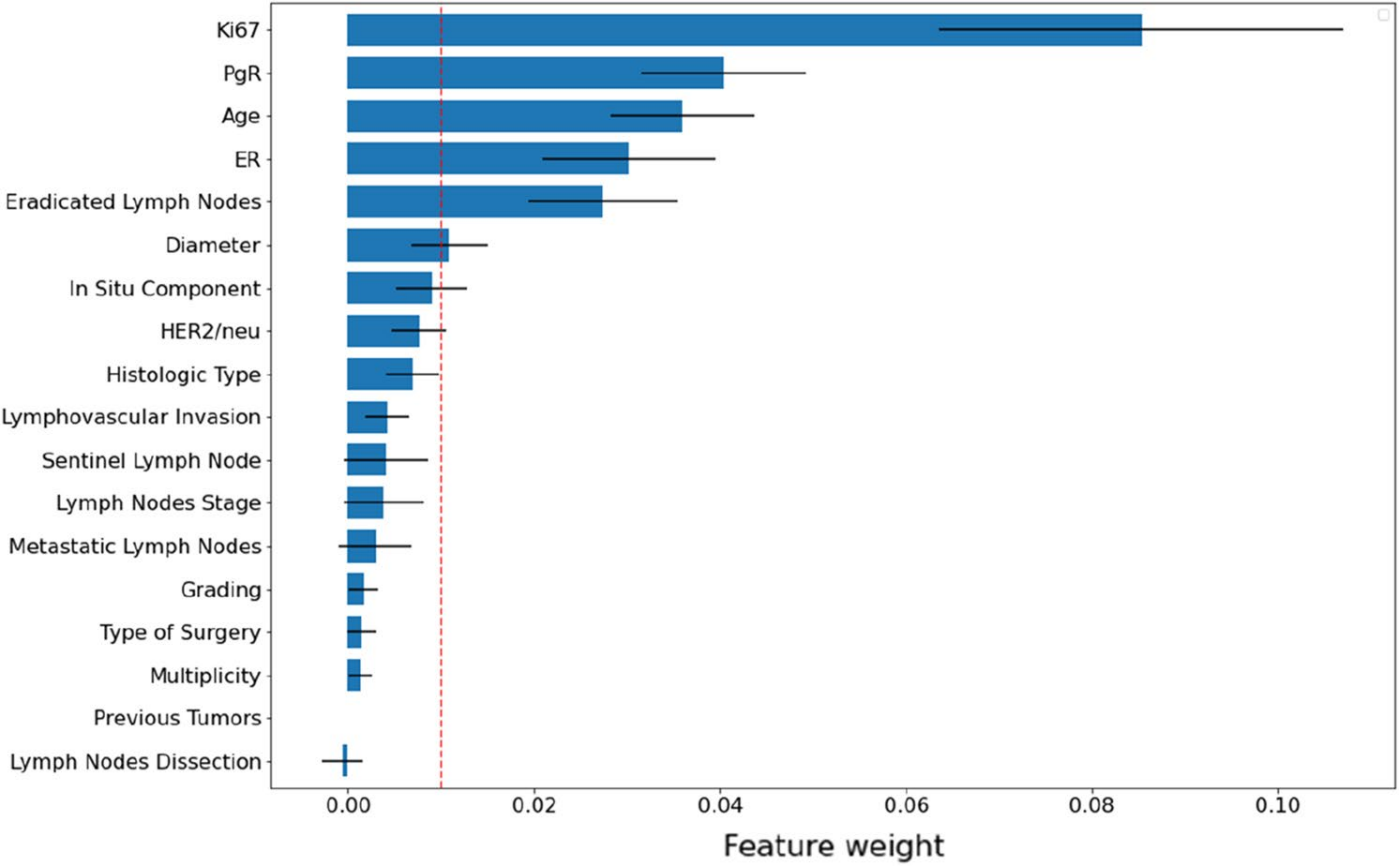
Random survival forest features importance averaged over 20 rounds of fivefold cross-validation. Error bars correspond to standard deviation and the red dashed line represents the imposed threshold for the mean feature weight.

Random survival forest feature importance is resumed in Fig. 1. Their calculation is nested in the 20 rounds of fivefold cross-validations to avoid any influence imposed by a single evaluation with a fixed training set. To take into account the included statistical variation, each feature weight is described by its average and standard deviation. We select those features characterized by a weight greater than 0.01, thus yielding: Ki67, PgR, age, ER, eradicated lymph nodes and diameter.

**Figure 2:**
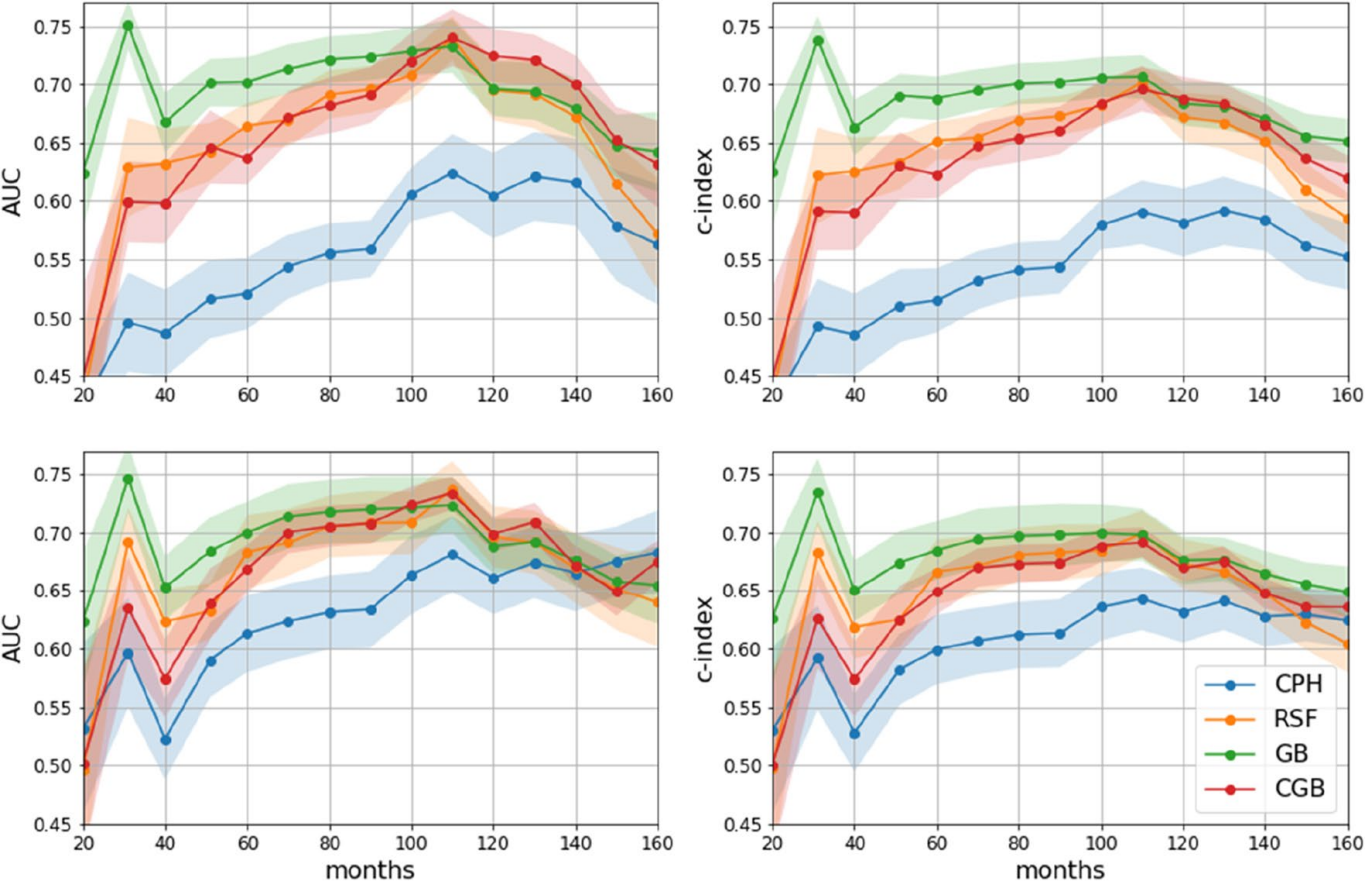
Description of the time behavior of the performance metrics, area under the ROC curve (AUC) and concordance index (c-index). Top panels are referred to the exploitation of the whole features set, while the bottom ones exploit just selected features. Solid lines join the mean points evaluated every 10 months, while shaded regions correspond to standard deviations.

The ability of machine learning survival algorithms to model data high dimensionality with respect to the classical CPH regression^18^ is shown by comparing the time behavior of the considered metrics (see Appendix B) when all features are included (N = 18) or just the six selected ones. In Fig. 2, the upper panels are related with the first case, while the lower panels to the latter one. At 5 and 10 years, time period usually considered for follow-up in clinical practice, the performances of the CPH model are on average lower than those of machine learning models by at least 10 percentage points, when all the features are considered. However, when just a subset of features is considered, consisting in the most important ones, the average difference in performance is halved, signaling a better condition for the CPH model, while the performance of machine learning survival models tends to remain unchanged with respect to the number of features involved. In order to define a parsimonious model, i.e. to use just the right number of predictors needed to explain the model well, the following analyses will be carried out on the results obtained by considering the selected subset of features.

The sensitivity and specificity balanced performance (see Fig. 6 in Appendix C) corresponding to the 5 years time frame are equal to 0.62–0.65 for the three machine learning survival models, while the much lower one of CPH shows approximately 0.55 for the same balanced metrics pair. If we consider 10 years after the first BC, the CPH model still shows the lowest performance, while the machine learning survival models RSF and GB are characterized by a balanced aforementioned metric pair equal to 0.63–0.65, while CGB emerges as the best performing classifier with 0.67 for the balanced sensitivity and specificity pair.

**Figure 3:**
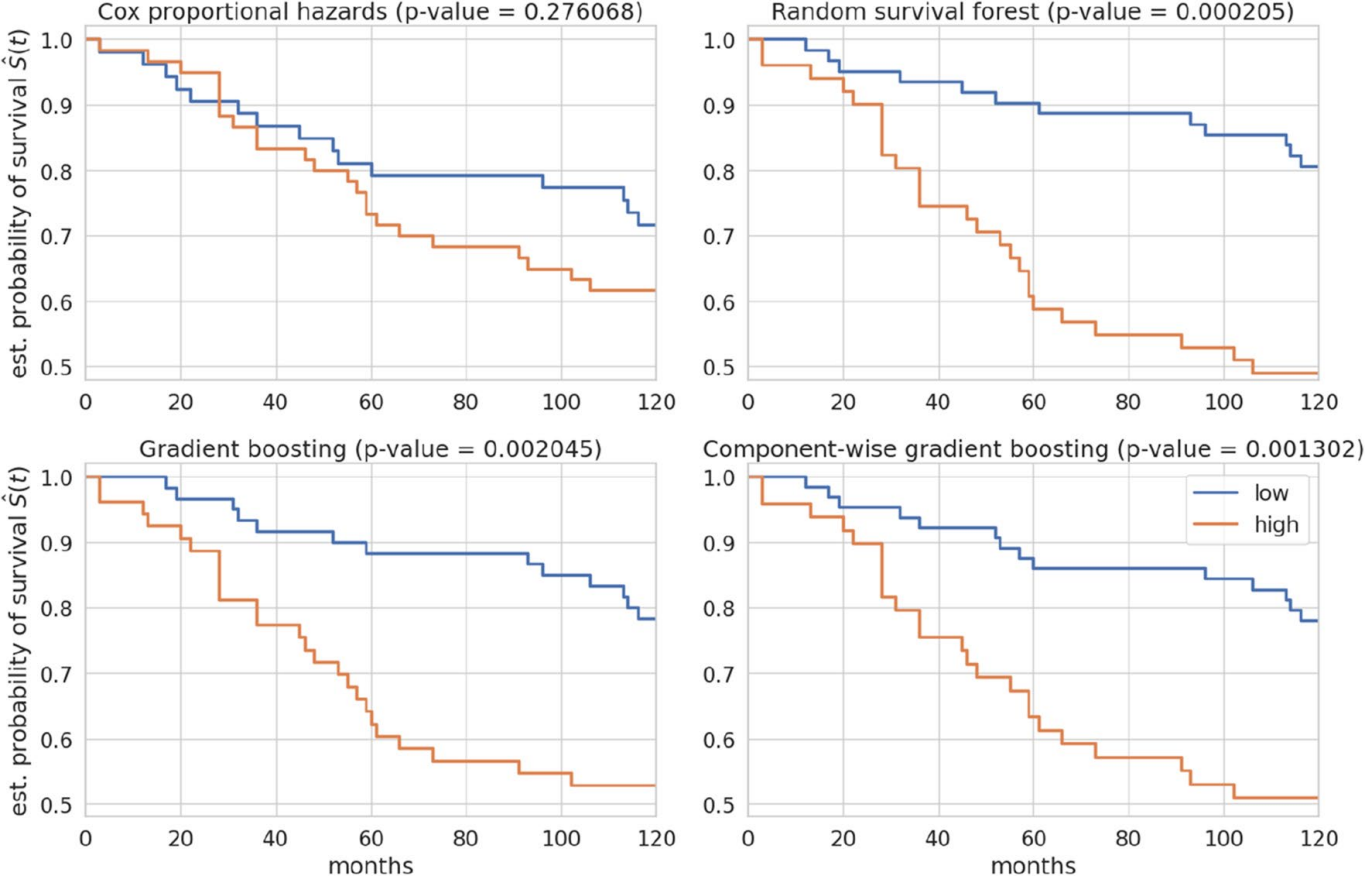
Representation of Kaplan–Meier survival probability for the patients stratification in high and low risk implemented by each model corresponding to 10 years after the first BC diagnosis.

Once the 10 years time frame is kept fixed, we establish a threshold for each model according to the median of the ones selected by the Youden index optimization (see Fig. 6 in Appendix B). The average score of each patient over the 20 rounds is then adopted to assign each case to a high or low risk category. These strata are further characterized by means of the Kaplan–Meier curves shown in Fig. 3. We reported in the descriptive table of survival estimation models in the supplementary materials (see Fig. 6). The *p* values confirm that CGB implements the best discrimination between high or low risk patients, while CPH is much less efficient than the machine learning survival models.

### Correlation with EndoPredict® scores

The similarity measure of our risk estimation with the one predicted by EP is evaluated over an independent test consisting of 27 patients. The last step of our preliminary study consists in the calculation of the Pearson correlation coefficients between the hazards (see Appendix A) estimated by our best performing CGB survival curves at 10 years after the first BC diagnosis and the risk scores provided to our institution by the EP software exploiting genetic data.

**Figure 4:**
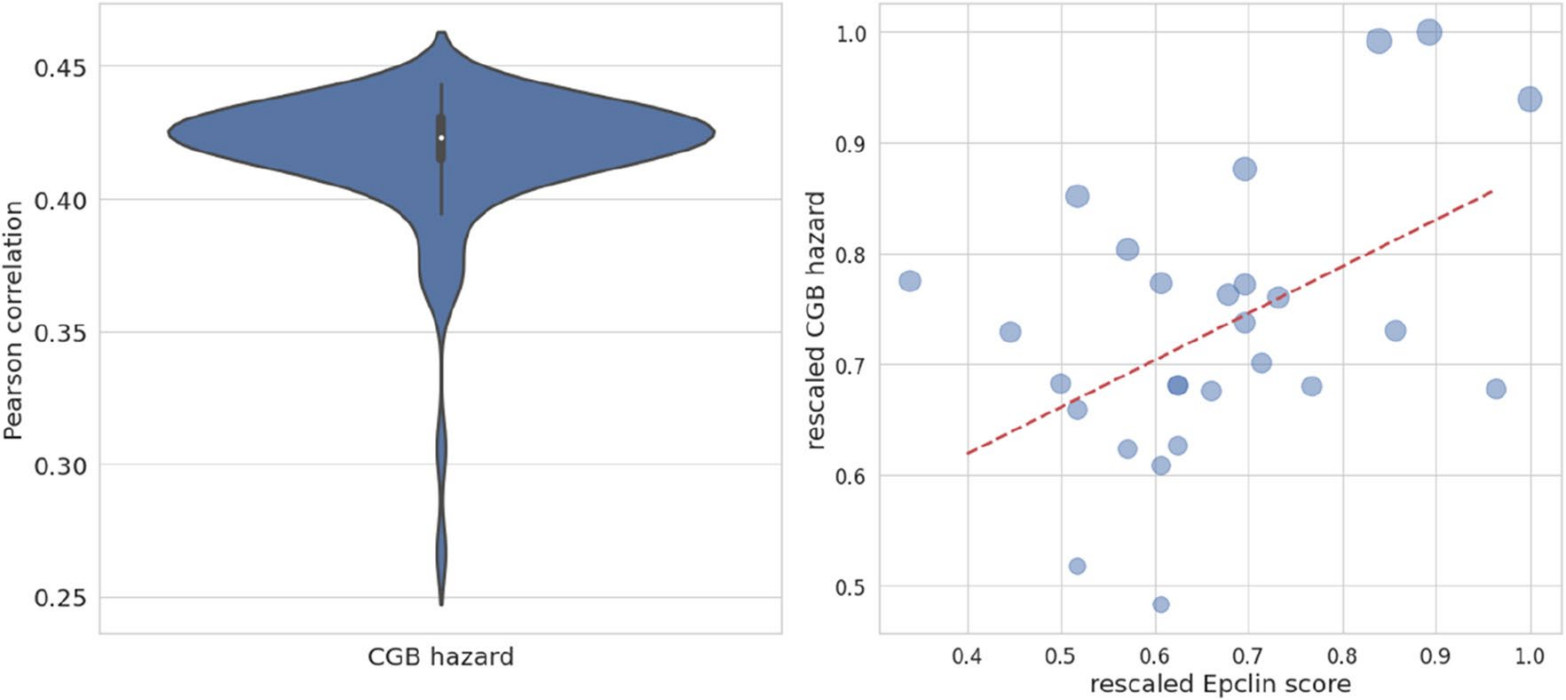
Representation of Pearson correlation statistics. In the left panel the distribution of correlations between the hazards yielded by the best performing CGB survival curves at 10 years and EP scores; In the right panel a bubble plot of scores and hazards in a rescaled version with respect to their maximum values, where each bubble ray measures the standard deviation over the 20 rounds, while the dashed red line slope is equal to the median value in the left panel.

A scores statistics for the separate dataset is obtained by testing the sample set of 27 patients on 20 rounds fivefold cross-validation for the model trained on 145 patients, such that a variation in the training is obtained to gain a wider statistic. The hazard value corresponding to 10 years after the first breast cancer diagnosis is then deduced, whose overall correlation statistics is shown in Fig. 4. The violin plot in the left panel is characterized by a sufficient stability around the median value, imposing the slope of the trend line in the bubble plot of the right panel.

The performance of EP declared by the authors in terms of c-index is equal to 0.753 for the prediction of distant recurrences within 10 years^5^. As emerged from our results, CGB shows the highest correlations, equal in average to 0.42, with respect to the remaining survival models, instead resulting in average uncorrelated. We underline that such correlations are time independent by definition for CGB, GB and CPH, because the corresponding hazard functions assume time independent parameters, such that features and time are independent variables.

## Discussion

The high recurrence rate characterizing BC patients has prompted to the adoption of post-operative treatments, including adjuvant chemotherapy. At the same time, the risk of overtreatment in this patient population has supported the development of tools able to perform a proper risk–benefit assessment and to guide the “decision-making” process^1,2,7^. The role of molecular data has become increasingly important in guiding therapeutic decisions in this setting. Nevertheless, there is the urgent need to explore novel reliable and less expensive prognostic tools. Particular attention deserver hormone-responsive, HER2 negative BC patients for which the prescription of an adjunctive chemotherapy hormone therapy is often highly doubtful. Recently, the genomic tests play a key rule into assess the benefit provided by the addition of chemotherapy, but are very expensive and their cost-effectiveness needs to be neglected. In this paper, we proposed a machine learning approach to estimate disease free survival.

To date, a plethora of predictive models have been developed to estimate disease-free survival with respect to recurrence breast cancer by solving a classification task^12,24,25^ or focusing on survival^12,26^. However, it is known that, despite not in common cases, anticancer drugs can cause second tumors, correlated with chemotherapy^27^. Therefore, recently, in the adjuvant clinical trial setting for breast cancer, experts proposed to adopt only one term, that is Invasive Disease-Free Survival, to refer to composite events, such as local and distant recurrence, contralateral invasive breast cancers, second primary tumors and death^28^. Recent works on survival model for invasive events prediction and its variants have been freshly proposed^29,30^ and were based on the exploitation of patients’ characteristics related to demographics, diagnosis, pathology and therapy. Among these, machine learning algorithms represent a novel, promising tool.

The usefulness of survival analysis inspired by machine learning algorithms is currently assessed by interdisciplinary studies, because of its improved ability to take into account high-dimensional data with respect to classical methods^18^. Such new approaches include a much higher complexity which require otherwise an accurate feature selection^4^.

Our preliminary study aims to evaluate the potential of machine learning models trained on commonly clinical features for predicting of IDEs for patients with endocrine-positive and HER2 negative BC. This tool, which has been built starting from information frequently collected in clinical practice, could replace the genomic profiling tests, notoriously more expensive in terms of application times and costs, when their application is not available^4,5^. In this preliminary work, we have compared three machine learning survival models with the classical approach, i.e. Cox proportional hazards regression, to predict IDES endocrine-positive and HER2 negative BC and, thus, identify low- and high-risk patients.

The c-index obtained within the same time frame by CGB, GB and RSF is stable with or without feature selection at approximately 0.67–0.68 in average. Considering that EP test declares a c-index equal to 0.753^5^, the obtained preliminary results including only clinical determinants are encouraging. We subsequently verified on an independent subset of patients who performed EP tests, whether the decision suggested by the model we trained was in agreement with the result of the EP test. Even if the latter takes into account genetic information not included in our survival models, we observe a sufficient similarity of the risk estimation for an invasive disease event corresponding to 10 years after the first BC, as measured by Pearson correlation. Indeed, the proposed model showed a significant agreement with the result of the EP test meaning that clinical features, if properly elaborated, could express part of the information expressed by the genomic test. However, the main advantage is the much cheaper and already available data exploited in our scheme, providing a sufficient information as measured by the correlation similarity.

If we consider the time period comprised between 5 and 10 years, a stable behavior of the mean performances emerges for the machine learning survival models. Moreover, they shown significantly higher risk estimation performance than the classical Cox model.

In addition, the machine learning survival models have shown a significant ability to predict the IDEs in both early and late periods (5 and 10 years, respectively), to accurately discriminate patients at low or high risk, and to detect a large risk patient group with positive outcome after 10 years with only 5 years of endocrine therapy.

To the best of our knowledge, studies aimed at developing a predictive model of IDFS for patients with endocrine-positive and HER2 negative BC are lacking. Therefore, we believe that a comparison between our results with those obtained with more generic state-of-the-art models that estimate overall survival or ides but trained on heterogenous populations, can be mistaking. What we consider interesting instead is the comparability of the forecast results of the survival disease carried out with genomic tests, as previously discussed. Another gene signature assay called Pam50 (Prosigna®) adopting 50 genes and engineered for distant recurrences declares a c-index related with the 3 years time frame equal to 0.72^31^, a value which is comparable with our performances.

Although the general performance does not yet allow a clinical application of the model, the experimental results encourage future developments aimed at introducing features of a different nature. Therefore, our hypothesis is that it is possible to define a machine learning model trained on data commonly collected in clinical practice that could accurately surrogate the genomic test, finally, reducing the cost of healthcare without compromising patient care, and significantly impacting clinical governance.

Further developments will be focused on the inclusion of radiomic features^32,33^ as well as some radiologic indices extracted from clinical reports. Indeed, the inclusion of imaging data is investigated in radiogenomics to reduce costs of genetic tests, thus proving an information resource that has to be comprised in a high-dimensional setting.

The usage of structured electronic health records is limited to relatively small datasets, while recent investigations are exploiting natural language processing to take free-text clinic notes as input^34,35^. Moreover, for the models adopted in survival analysis, much general hypotheses have been formulated to take into account the time dependence of regression coefficients, thus giving up the assumed independence of features in factorized hazard functions^36–39^, which is partly observed just for RSF among the implemented machine learning survival models.

## Materials and methods

### Survival analysis for risk estimation

In this work, we propose the application of machine learning survival models to evaluate the risk of an invasive disease event for each patient. Such models are formulated in Appendix A, implemented by using the Python package scikit-survival (v. 0.17.1) and listed as follows:

1. Random survival forest (RSF);
2. Gradient boosting (GB);
3. Component-wise gradient boosting (CGB).

A comparison of these machine learning methodologies with the well-known Cox proportional hazards (CPH) are performed.

The selection of most important features is executed by means of the Python package eli5 (v. 0.11), which provides a way to compute feature importance by measuring how concordance index (c-index) decreases when a feature is not available. In the survival framework, the remotion of the relationship of a certain feature with the survival time is executed by random shuffling of its values: the weight of each feature is quantified by the drop on average of the c-index^40^. The performance metrics are estimated in a time dependent approach^22^ and they are obtained by adopting both the whole set of features and just those characterized by a sufficient weight in the aforementioned importance evaluation. To understand the variation in time of some metrics exploited to assess the inferential power of a survival model, we have to rephrase it as a classifier yielding a time varying score equal to the complement to one of the survival probability

1 $$F_{i} \left( t \right) = 1 - S_{i} \left( t \right),$$

with $i = 1, \ldots ,M$ labelling each patient in the sample, with $M$ equal to the total number of patients. The observed event time is

2 $$Z_{i} = {\text{min}}\left\{ {T_{i} ,C_{i} } \right\},$$

where $T_{i}$ denotes the time of invasive disease onset and $C_{i}$ the censoring time. In this way a time dependent disease status $D_{i} \left( t \right)$ takes value 0 until $t < T_{i}$, while it shifts to 1 afterwards^22^. The censored patients, not showing any event, are removed when $t > C_{i}$, as shown in Table 2 starting from 20 months to avoid too unbalanced data sets.

**Table 2:** Summary of the sample labels variation in the time period comprised between 20 and 160 months.

Invasive disease events	7	12	17	20	27	29	30	30	33	35	38	39	41	43	44
Control cases	136	130	125	121	112	109	107	103	91	82	75	64	47	31	20
Months	20	31	40	51	60	70	80	90	100	110	120	130	140	150	160

The precise formulation of the adopted metrics consisting in the time dependent version of the area under the receiver operating characteristic (ROC) curve (AUC) and c-index is presented in Appendix B. These quantities are able to describe time by time how the survival models capture the patient’s status behavior with respect to the used features. The optimization of the available parameters was implemented on ReCaS datacenter^41^. Later on, the analysis considered just the fixed time frame corresponding to 5 and 10 years after the first BC diagnosis. The research of an optimal threshold balancing sensitivity and specificity is based on the Youden index^4^, whose maximization drives the solution achievement. To measure the efficiency of our scheme in estimating patients risks, the comparison with EP scores associated with a separate set of 27 patients is implemented. Such patients are endowed with the feature subset selected in the described procedure.

### Institutional review board statement

Institutional Review Board Statement: The study received approval from the Scientific Board of Istituto Tumori “Giovanni Paolo II”—Bari, Italy and was carried out in accordance with the Declaration of Helsinki’s standards. The authors affiliated to the Istituto Tumori “Giovanni Paolo II” RCCS, Bari are responsible for the views expressed in this article, which do not necessarily represent the ones of the Institute.

### Informed consent statement

Written informed consent for this study was waived by the Scientific Board of Istituto Tumori “Giovanni Paolo II”—Bari, Italy due to retrospective study.

## Supplementary Information

Supplementary Information.

## Data Availability

The raw data supporting the conclusions of this article will be made available by the corresponding author, without undue reservation.
