# Supplementary material for: Machine learning survival models trained on clinical data to identify high risk patients with hormone responsive HER2 negative breast cancer
Source: Sci Rep. 2023 May 26;13:8575. doi: 10.1038/s41598-023-35344-9 (PMC10220052; doi:10.1038/s41598-023-35344-9)
Supplement: Supplementary file 1 — Supplementary Information. [file 41598_2023_35344_MOESM1_ESM.docx]

**SUPPLEMENTARY MATERIALS**

**Appendix A**

The survival analysis considers a sample composed by $M$ individuals, with $n$ failure events at times $t_{(1)}<t_{(2)}<\ldots<t_{(k)}$ and multiplicity $m_{(j)}$ such that $\sum_{j=1}^{k} m_{(j)}=n$. By denoting with $R(t_{\left( j \right)})$ the set of individuals at risk at time $t_{\left( j \right)}$, in a number (set cardinality) $|R(t_{\left( j \right)})|=r_{(j)}$, it is possible to define the hazard function

| $h\left( t \right)=\sum_{j=1}^{k} h_{t_{\left( j \right)}}\delta\left( t-t_{\left( j \right)} \right)=\sum_{j=1}^{k} \frac{m_{\left( j \right)}}{r_{\left( j \right)}}\delta\left( t-t_{\left( j \right)} \right)$, | (A1) |
| --- | --- |

as represented in Figure 5 with an example. The survival function is defined in terms of the non decreasing cumulative hazard function (CHF)

| $S\left( t \right)=exp( -H(t))$, with $H\left( t \right)=\int_{0}^{t} h(s)ds$, | (A2) |
| --- | --- |

as a monotonically decreasing function.

**Figure 5.** The representation of an hazard function.

The Cox proportional hazards regression is build as an inferential model adopting the following expression for the hazard function

| $h\left( t\vert\boldsymbol{x}_{i} \right)=h_{0}\left( t \right)\exp(\boldsymbol{\beta⦁} \boldsymbol{x}_{i})$, | (A3) |
| --- | --- |

with $\boldsymbol{\beta}$ and $\boldsymbol{x}_{i}$ two vectors with $N$ components in scalar product, the latter representing the features of the $i$-th patient, while $h_{0}\left( t \right)$ is called baseline hazard function for $\boldsymbol{x}_{i}=0$. Statistical inference is achieved by means of likelihood over a training subsample related with the probability that a failure at time $t_{(j)}$, conditionally on the risk set $R(t_{\left( j \right)})$

| $P\left( T=t_{\left( j \right)} \vert T\geq t_{\left( j \right)} \right)=\frac{P(T=t_{\left( j \right)})}{P(T\geq t_{\left( j \right)})}=\frac{h\left( t\vert\boldsymbol{x}_{(j)} \right)}{\sum_{l\in R\left( t_{\left( j \right)} \right)} h\left( t\vert\boldsymbol{x}_{l} \right)}$, | (A4) |
| --- | --- |

is observed given the individual $\boldsymbol{x}_{(j)}$, thus implying in the assumption of statistical independence

| $L\left( \boldsymbol{\beta} \right)=\prod_{j=1}^{M} \left[ \frac{\exp(\boldsymbol{\beta⦁} \boldsymbol{x}_{(j)})}{\sum_{l\in R\left( t_{\left( j \right)} \right)} \exp(\boldsymbol{\beta⦁} \boldsymbol{x}_{l})} \right]^{\delta_{j}}$, | (A5) |
| --- | --- |

where a censoring indicator $\delta_{i}$ is introduced. It takes value 1 if the disease occurs and 0 otherwise, such that each patient presenting $\delta_{i}=1$ for the disease establishment at a certain time is endowed with ${Z_{i}=T}_{i}$, alternatively ${Z_{i}=C}_{i}$, as expressed by Eq. (2) in the main text. The maximum likelihood problem leads to the log-likelihood

| $\mathcal{l}\left( \boldsymbol{\beta} \right)=\sum_{j=1}^{M} \delta_{j}\boldsymbol{(\beta⦁} \boldsymbol{x}_{(j)}\boldsymbol{)-}\sum_{j=1}^{M} \delta_{j} log(\sum_{l\in R\left( t_{\left( j \right)} \right)} \exp(\boldsymbol{\beta⦁} \boldsymbol{x}_{l}))$, | (A6) |
| --- | --- |

whose solution yields the parameters estimator $\hat{\boldsymbol{\beta}}\boldsymbol{=}\mathrm{argmax}_{\boldsymbol{\beta}}\mathcal{(l}\left( \boldsymbol{\beta} \right))$ [42].

This inferential approach to survival analysis has been extended to include machine learning survival models, in particular involving ensemble methods. Among the latter random forest plays an important role as representing the conventional algorithm and for this reason we used the random survival forest [14].

An ensemble method considers multiple models, each one associated with a bootstrap sample. For the specific case of random survival forest there are many survival trees, where at each node some candidate features are randomly selected. Then the node is split using the candidate variable that maximizes survival difference between daughter nodes. When each tree has grown up to full size, the CHF is evaluated in each terminal node. Each of the latter is labeled by $f$, including $M\left( f \right)$ individuals, $m_{l,f}$ number of events and $r_{l,f}$ individuals at risk at time $t_{l,f}$, such that

| $H_{f}\left( t \right)=\sum_{t_{l,f}\leq t} \frac{m_{l,f}}{r_{l,f}}$, | (A7) |
| --- | --- |

is the CHF estimate per node. If $\boldsymbol{x}_{i}$ falls down in the terminal node $f$, we have $H\left( t | \boldsymbol{x}_{i} \right)=H_{f}(t)$ and by denoting $H_{b}\left( t | \boldsymbol{x}_{i} \right)$ the CHF of a tree in the $b$-th bootstrap sample, the ensemble CHF is

| $H_{e}\left( t \vert\boldsymbol{x}_{i} \right)=\frac{1}{B}\sum_{b=1}^{B} H_{b}\left( t \vert\boldsymbol{x}_{i} \right)$, | (A8) |
| --- | --- |

where $B$ is the total number of trees [14].

The gradient boosting approach is based on the minimization of a loss function $\mathcal{L}\left( \boldsymbol{\beta} \right)$, that we choose as the negative of $\mathcal{l}\left( \boldsymbol{\beta} \right)$ in Eq. (A6), where the linear regression $\boldsymbol{\beta⦁} \boldsymbol{x}_{i}$ is replaced by a general regression function $\psi_{\boldsymbol{\beta}}(\boldsymbol{x}_{i})$ [15-17]. The latter has to be approximated by a sequence of functions $\varphi_{p}(\boldsymbol{x}_{i})$ in an iterative procedure with steps labeled by $p$ and adopting base learners $b(\boldsymbol{x}_{i}|\boldsymbol{\beta}_{p})$. A each step the gradient of the loss function is computed

| $U_{i}=\frac{\partial\mathcal{L}\left( \boldsymbol{\beta} \right)}{\partial\psi}\left. \right\vert_{{\psi=\varphi}_{p}(\boldsymbol{x}_{i})}$, | (A9) |
| --- | --- |

called working response or pseudo-response, because base learners will fit according to the following minimization

| $\boldsymbol{\beta}_{p}=\mathrm{argmin}_{\boldsymbol{\beta}}\sum_{i} \left( U_{i}-b(\boldsymbol{x}_{i}\vert\boldsymbol{\beta}) \right)^{2}$, | (A10) |
| --- | --- |

which leads to the update $\varphi_{p+1}\left( \boldsymbol{x}_{i} \right)=\varphi_{p}\left( \boldsymbol{x}_{i} \right)+\nu b(\boldsymbol{x}_{i}|\boldsymbol{\beta}_{p})$, with the chosen learning rate $\nu=0.1$. The component-wise gradient boosting adopts as base learners in Eq. (A10) a linear model, implementing the update of parameters according to just one feature, which minimizes the difference with the pseudo-response vector.

**Appendix B**

The patient subset endowed with $\delta_{i}=1$ is retained for all times, while those patients with $\delta_{i}=0$ can be removed for $t>C_{i}$. The time variation for sensitivity and specificity is related with the introduction of a threshold $c$, such that

| $sens\left( c,t \right)=P(F_{i}\left( t \right)>c\vert D_{i}\left( t \right)=1)$, $spec\left( c,t \right)=P(F_{i}\left( t \right)\leq c\vert D_{i}\left( t \right)=0)$, | (B1) |
| --- | --- |

where $P$ stands for a conditional probability.

| 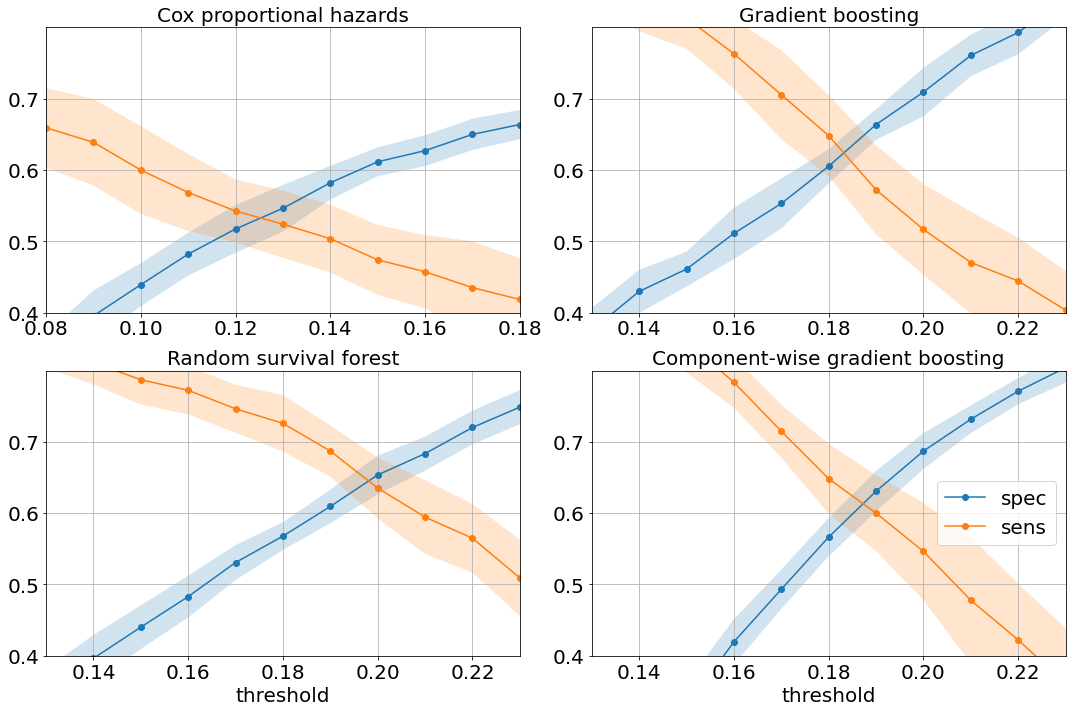 | 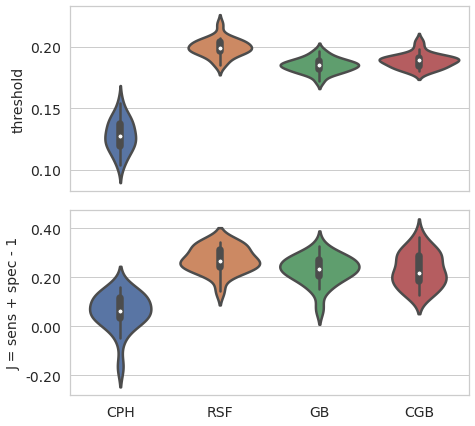 |
| --- | --- |
| (**a**) | (**b**) |
| 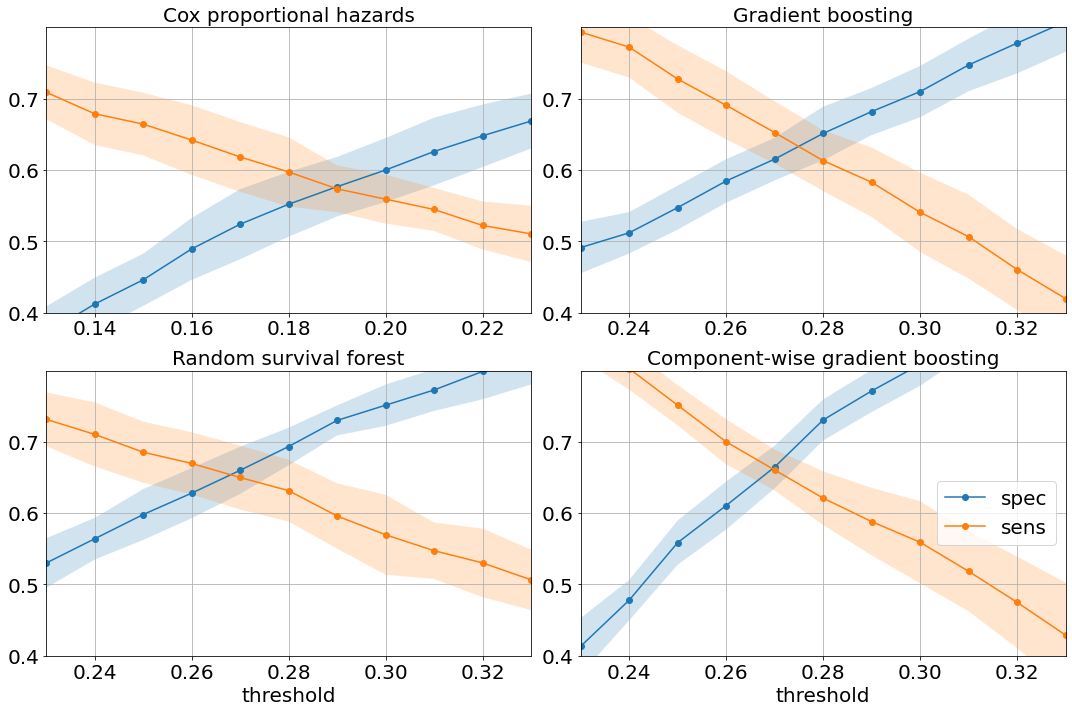 | 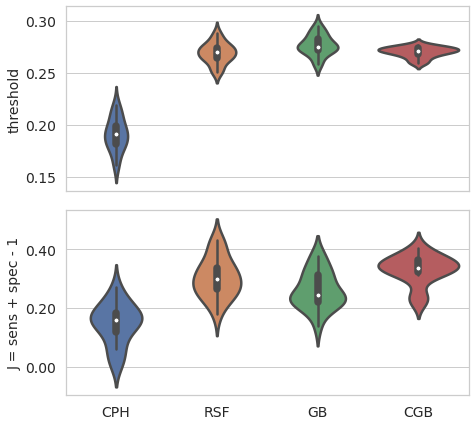 |
| (**c**) | (**d**) |

**Figure 6.** Representation of the optimal threshold for the four classifiers framed at 5 years after the first BC diagnosis in the upper panels and 10 years in the lower ones. (**a-c**) The evaluation of sensitivity and specificity for equally spaced thresholds values, where solid lines join points representing the mean values with standard deviation expressed by the shaded region; (**b-d**) Description of the Youden index statistics in the lower subpanel, with associated thresholds in the upper one verifying those characterized in panel (**a-c**).

Once these quantities are introduced, it is possible to define the first metric we are going to use

| $AUC(t)=\int_{0}^{1} sens\left( c,t \right)d[1-spec\left( c,t \right)]$, | (B2) |
| --- | --- |

namely the area under the ROC curve, where $d\left[ 1-spec\left( c,t \right) \right]=\frac{\partial\left[ 1-spec\left( c,t \right) \right]}{\partial c}dc$ denotes the differential [22].

The concordance index is expressed by adopting the time varying score defined in Eq. (1) of the main text

| $c-index\left( t \right)=P\left( F_{i}\left( t \right)>F_{j}\left( t \right) \vert T_{i}<T_{j} \right).$ | (B3) |
| --- | --- |

**Appendix C**

The research for balanced sensitivity and specificity metrics by choosing a certain score threshold is well captured by the Youden index maximization. In Figure 6, we show the study aimed to identify the optimal threshold, by considering the chosen features subset it is less clear what is the best performing model as shown in Figure 2. Once the optimal threshold is established in panels (a-c) of Figure 6, the sensitivity and specificity pair of machine learning survival models shows the highest mean values in panels (a-c). The same thresholds statistics is verified by the Youden index represented in panel (b-d), whose values confirm the aforementioned balanced metrics pair, expressing the classifier ability to capture true positive and true negative patients respectively (see Appendix B). The CPH survival model shows a much lower value of the aforementioned metrics. The increased values for the optimal thresholds is proportional with the ratio of invasive disease events over the total number of patients shown in Table 2 corresponding to 60 and 120 months.
